# The Effect of Pantoprazole on Midline Closure in Early Chicken Embryos: An Experimental Study

**DOI:** 10.7759/cureus.97084

**Published:** 2025-11-17

**Authors:** Hasan Turkoglu, Muhammet Kirkgecit, Recai Engin, Öykü Dila Gemci, Cem Kesilmez

**Affiliations:** 1 Department of Neurosurgery, Gaziantep City Hospital, Gaziantep, TUR; 2 Department of Neurosurgery, Elbistan State Hospital, Kahramanmaraş, TUR; 3 Department of Neurosurgery, Samsun University Faculty of Medicine, Samsun, TUR; 4 Department of Pathology and Laboratory Medicine, Elbistan State Hospital, Kahramanmaraş, TUR; 5 Department of Neurosurgery, Kahramanmaraş Sutcu Imam University, Kahramanmaraş, TUR

**Keywords:** embryo, neural tube defects, pantoprazole, pregnancy, teratogenicity

## Abstract

Pantoprazole is a proton pump inhibitor commonly used to treat gastroesophageal reflux disease (GERD) by reducing stomach acid production. Although widely used for gastrointestinal issues, its effects on embryonic development remain underexplored. This study investigated the impact of pantoprazole on neural tube closure using a chick embryo model. Fertilised chicken eggs were divided into groups and treated with varying doses of pantoprazole. All embryos were collected on the eighth day of incubation. Both macroscopic and microscopic examinations were conducted to assess developmental abnormalities. Macroscopic analysis revealed malformations, particularly in embryos exposed to medium and high doses of pantoprazole. These groups exhibited statistically significant developmental delays compared to controls. Microscopic examination further demonstrated that high-dose pantoprazole resulted in delayed vertebral lamina ossification and midline closure defects-key indicators of neural tube defects. Overall, the study concluded that high levels of pantoprazole exposure during embryonic development can lead to structural malformations and delayed growth. Specifically, neural tube defects were identified, indicating a risk to early neural development. These findings suggest that pantoprazole use during pregnancy may pose teratogenic risks and should be approached with caution. This research highlights the need for further studies on the safety of commonly used medications like pantoprazole during pregnancy, as their use may adversely affect embryonic development, particularly neural tube formation.

## Introduction

Neural tube defects (NTDs) are congenital malformations seen in about one in 1000 pregnancies globally, with prevalence ranging from two to 10 per 1000, depending on geographic factors [1]. Studies show that NTD frequency is higher in abortion samples. These defects, alongside congenital heart and genitourinary anomalies, are among the most common congenital malformations [2]. The first epidemiological data on NTDs were published by Record and McKeown in 1949, based on 930 cases of central nervous system malformations [3]. Further analysis indicates that NTD characteristics vary by ethnicity, geography, socio-economic factors, and the higher incidence of anencephaly in female fetuses of multiparous women. The etiology of NTDs is multifactorial [4], with recent studies highlighting the role of environmental factors, genetic predispositions, and folate deficiency. NTDs include malformations like encephalocele, anencephaly, spina bifida, and neuroenteric cysts [5]. These arise from disruptions during neurulation, a critical stage in central nervous system development [6]. Neurulation begins around 16 days post-ovulation, with epiblastic cells migrating along the primitive streak to form the neuroectodermal tissue [7]. This neural layer extends towards somite pairs, surrounded by mesoderm cells that form vertebrae and ribs. Neural and non-neural ectodermal cells interact to form the neural tube, which folds and fuses along the midline [6]. Recent research shows that neurulation occurs through progressive waves of closure, rather than from cranial to caudal [8,9].

Chicken embryonic development spans 46 stages, with focus on stages 44-48, especially up to stage 13, for studying neural development [10].

Pantoprazole, used for acid-related gastrointestinal disorders, has been linked to significant biochemical changes and adverse reactions, including pancytopenia, skin reactions, edema, nephritis, anaphylaxis, hyponatremia, hepatotoxicity, and hypomagnesemia [11,12]. This study investigates the effects of pantoprazole-containing drugs on chick embryo development, particularly midline closure.

## Materials and methods

Before starting the study, ethics committee approval was obtained from Kahramanmaraş Sütçü İmam University ethics committee with the decision number 2024/03 and date 25.10.2024. 

Experimental model and chemicals used

Broiler eggs, previously stored at 18°C to inhibit embryonic development, were obtained for the study (n=100). All embryos were at the same developmental stage. Pantoprazole, commercially sourced, was used for the experiment. A 70 mg pantoprazole capsule, intended for oral administration, was dissolved in 2 ml of 0.1 M dimethyl sulfoxide (DMSO), followed by vortexing, sonication, and dilution to the desired concentrations. The eggs were weighed and divided into five equal groups. The average egg weight was 50 g ± 2 g (n=10). A literature review established the maximum recommended dose of pantoprazole for humans as 70 mg/day (for a 70 kg individual) [13]. Dose calculations per person are as follows: 17.5 mg/day/70 kg = 0.2 mg/kg (0.01 mg/50 g egg); 35 mg/day/70 kg = 0.4 mg/kg (0.02 mg/50 g egg); 70 mg/day/70 kg = 0.8 mg/kg (0.04 mg/50 g egg).

Pantoprazole application and incubation process

Prior to conducting the experiment, initial analyses were performed on the incubator. The temperature, humidity, and repositioning time interval values were determined and recorded. The incubator was set to a humidity level of 50% (± 5%) and a temperature of 37°C (± 0.2°C). The incubator was programmed to rotate the eggs 45 degrees in the opposite direction every six hours.

On the day of the experiment, the eggs were cleaned with ethanol, and a 0.5 mm hole was created in the air sacs of the eggs using a needle. The volumes of carrier and drug, as outlined in Table 1, were then injected into each egg sac using a 26 G needle. Following the injection, the eggs were sealed with sterile tape and transferred to the incubator. Temperature and humidity levels were monitored twice daily.

**Table 1:** Experimental Model, Chemicals Used and Dosage Table for Groups This table summarizes the experimental grouping, the chemicals administered, and the corresponding dosages used in the study. Five groups were formed: a control group (no intervention), a vehicle group receiving dimethyl sulfoxide (DMSO) as solvent, and three experimental groups treated with pantoprazole at doses of 0.2, 0.4, and 0.8 mg/kg, respectively. All embryos received the same total injection volume (0.3 mL) to ensure uniform exposure conditions. The “extrapolation to human” column indicates the theoretical equivalent daily dose based on body weight, allowing for comparison with human pharmacological exposure levels. This table provides the dosing framework for assessing the dose-dependent embryotoxic potential of pantoprazole during early development. Note: The calculated volume of the carrier (DMSO) was determined as 0.02 ml for each experimental group. For this reason, it was taken as 0.02 ml for each egg in Group II. In order to provide the same injection volume in the experimental groups, dilution was made to 0.03 ml.

Group (n)	Explanation	Chemical	Dose	Volume	Extrapolation To Human
Group I (n=20)	Control	None	None	None	0 mg / day
Group II (n=20)	Vehicle	DMSO	0.02ml / egg	0.3 ml	0 mg / day
Group III (n=20)	Experiment	Pantoprazol	0.2 mg / kg 0.01 mg / egg	0.3 ml	17.5 mg / day
Group IV (n=20)	Experiment	Pantoprazol	0.4 mg / kg 0.02 mg / egg	0.3 ml	35 mg / day
Group V (n=20)	Experiment	Pantoprazol	0.8 mg / kg 0.04 mg / egg	0.3 ml	70 mg / day

After eight days, the embryos were harvested, and the chorioallantoic membranes, blood vessels, egg white, and yolk were carefully removed. The embryos were washed multiple times with saline solution before being dissected and examined under a dissecting microscope. Post-examination, the embryos were transferred to a formaldehyde solution for preservation. A day later, the embryos were re-examined for weight, head diameter, choriocaudal length, spina bifida, encephalocele, anencephaly, and other significant developmental malformations. The results of this examination are presented in Table 2.

**Table 2:** Number of Fertilized Eggs, Early Embryo Mortality, Developmental Retardation and Measurement Results of All Embryos by Groups This table presents the distribution of fertilized eggs, early embryonic deaths, developmental retardation, and morphometric parameters among experimental groups. Pantoprazole exposure at increasing doses (0.2 mg/kg, 0.4 mg/kg, 0.8 mg/kg) was associated with a dose-dependent trend toward higher embryonic mortality and developmental delay.  Data are expressed as mean ± standard deviation. Differences among groups were analyzed using one-way ANOVA, and F statistics with corresponding p values are shown in the last two columns. When significant differences were detected (p < 0.05), Tukey’s post hoc test was used to determine pairwise group differences, represented by superscript letters (a, b, c, d, etc.). (Abbreviations: cm, centimeter; gr, gram; F, F-test statistic from ANOVA). Note: There is a significant difference between the same exponential letters

Group	Explanation	Fertilized egg	Early embryo death	Severe Retardation	Number of measured embryos	Mean Cranio caudal lenght (cm)	Mean Head Diameter (cm)	Mean Abdomen Diameter (cm)	Mean Embryo Weight (gr)
Group I	Control	18	2	0	18	2.78^a,b,c,d^	3.32^a,b,c^	2.42^ a,b,c,d^	0.98
Group II	Vehicle	19	1	0	19	2.25^a,e,f,g^	2.73^a,d,e,f^	1.96^ a,e,f,g^	0.63
Group III	0.2 mg/kg	17	3	2^a^	17	2.41^b,e,h,i^	3.26^d,g,h^	2.18^ b,e,h,i^	0.73
Group IV	0.4 mg/kg	15	5	2^b^	15	3.09^c,f,h^	3.78^b,e,g^	2.63^c,f,h^	1.19
Group V	0.8 mg/kg	15	5	7^a,b^	15	3.15^d,g,i^	3.81^c,f,h^	2.64^d,g,i^	1.36
p		0.313	0.316	0.002	0.313	0	0	0	<0.05
F (ANOVA)		1.775	1.783	4.378	1.775	9.461	8.457	9.175	4.376

Statistical analysis

The mean ± standard deviation values of the continuous variables used in the study were determined first. Then, parametric statistical methods were used on the data used here. It was determined that the groups here showed normal distribution. For this reason, the analysis process was carried out using Shapiro-Wilk tests. Then, one-way ANOVA test was performed and post hoc multiple Benferroni tests were used to compare the smallest differences here. The Mann-Whitney test and Kruskal-Wallis test were applied between the groups showing abnormal distribution here and the analysis was carried out. The p value <0.05 was considered statistically significant. The statistical analyzes were performed on SPSS Statistics version 26.0 (IBM Corp., Armonk, NY, USA).

## Results

Five experimental groups were established, including one control, one vehicle, and three pantoprazole-treated groups at doses of 0.2, 0.4, and 0.8 mg/kg (Table 1). All groups received the same total injection volume (0.3 ml), with DMSO used as the solvent to standardize administration. The experimental design allowed dose-dependent evaluation of pantoprazole’s effects on early embryonic development and midline closure.

Sixteen eggs were determined to be non-fertilized (Group I: two eggs; Group II: one egg; Group III: three eggs; Group IV: five eggs; Group V: five eggs). Developmental retardation was observed in 11 eggs (Group III: two eggs; Group IV: two eggs; Group V: seven eggs). The staging system used for this study was based on Hamburger and Hamilton [14].

In Group I, all embryos were found to be at the 33rd-34th incubation stage according to the Hamburger and Hamilton classification (1951). The average craniocaudal length of the embryos in Group I was 2.789 cm (range: 2.0 cm to 3.2 cm); the mean weight was 0.986 g (range: 0.65 g to 1.34 g); the mean head diameter was 3.326 cm (range: 2.3 cm to 4.2 cm); and the mean abdominal diameter was 2.425 cm (range: 2.3 cm to 2.9 cm) (Table 2). In contrast, the values for the delayed embryos were as follows: craniocaudal length 2.0 cm, mean weight 0.65 g, mean head diameter 2.2 cm.

There were two early embryo deaths in Group I, one in Group II, three in Group III, five in Group IV, and five in Group V (the Hamburger and Hamilton stage was not confirmed for these embryos). As a result, measurements could not be obtained for these embryos. All remaining embryos were determined to be in the Hamburger and Hamilton stage at the 33rd to 34th stage of incubation.

In Group II, the mean craniocaudal length of all embryos was 2.225 cm (range: 1.8 cm to 3.3 cm); the mean weight was 0.637 g (range: 0.65 g to 0.72 g); the mean head diameter was 2.739 cm (range: 2.4 cm to 2.9 cm) was 1.965 cm (range: 1.8 cm to 2.2 cm).

In Group III, embryos were found to be suitable for the 33rd-34th incubation stage according to the Hamburger and Hamilton staging system. In this group, the craniocaudal length of all embryos was 2.414 cm (range: 2.3 cm to 2.8 cm); the mean weight was 0.736 g (range: 0.65 g to 0.85 g); the mean head diameter was 3.269 cm (range: 3.0 cm to 3.6 cm).

In Group IV, five embryos exhibited early death, which prevented measurements for these embryos (Table 3). The remaining embryos in this group did not correspond to the expected stage of development at the 35th-36th incubation stage according to the Hamburger and Hamilton staging system. In this group, the craniocaudal length of all embryos was 3.098 cm (range: 2.9 cm to 3.2 cm); the average weight was 1.197 g (range: 0.98 g to 1.93 g); the average head diameter was 3.784 cm (range: 3.2 cm to 3.9 cm).

**Table 3:** Defects observed in the experimental groups This table summarizes the distribution of structural developmental defects observed in chick embryos across all study groups. Parameters include lack of development, developmental delay, and neural tube status (open or closed). Neural tube opening was observed only in the higher-dose groups (0.4 mg/kg and 0.8 mg/kg), while complete closure predominated in the control and low-dose groups. Categorical variables (no development, developmental delay, neural tube open/closed) were compared using the Chi-square (χ²) test. The corresponding p values and χ² statistics are presented in the last two columns. Superscript letters (a, b, c, etc.) denote significant inter-group differences (p < 0.05). (Abbreviations: NTO, neural tube open; NTC, neural tube closed; χ², Chi-square test statistic). Note: There is a significant difference between the same exponential letters

Group	No development	Development delay	Neural tube open	Neural tube closed
Control	2	0^a,b,c^	0	18^a^
Shum	1	0^d,e,f^	0	19^b^
Px	3	2^a,d,g^	0	15^c^
Px2	5	2^b,e,h^	1	12^d^
Px4	5	7^c,f,g,h^	1	7^a,b,c,d^
p	.313	.002	.548	.000
χ² (Chi-square)	4.760	16.920	3.060	23.510

In Group V, significant developmental delay was observed in seven embryos. Early death was noted in five embryos, preventing measurements. For the remaining embryos in this group, the craniocaudal length was 3.158 cm (range: 2.9 cm to 3.3 cm); the average weight was 1.365 g (range: 1.20 g to 1.45 g); the average head diameter was 3.814 cm (range: 3.6 cm to 4.0 cm).

**Figure 1:**
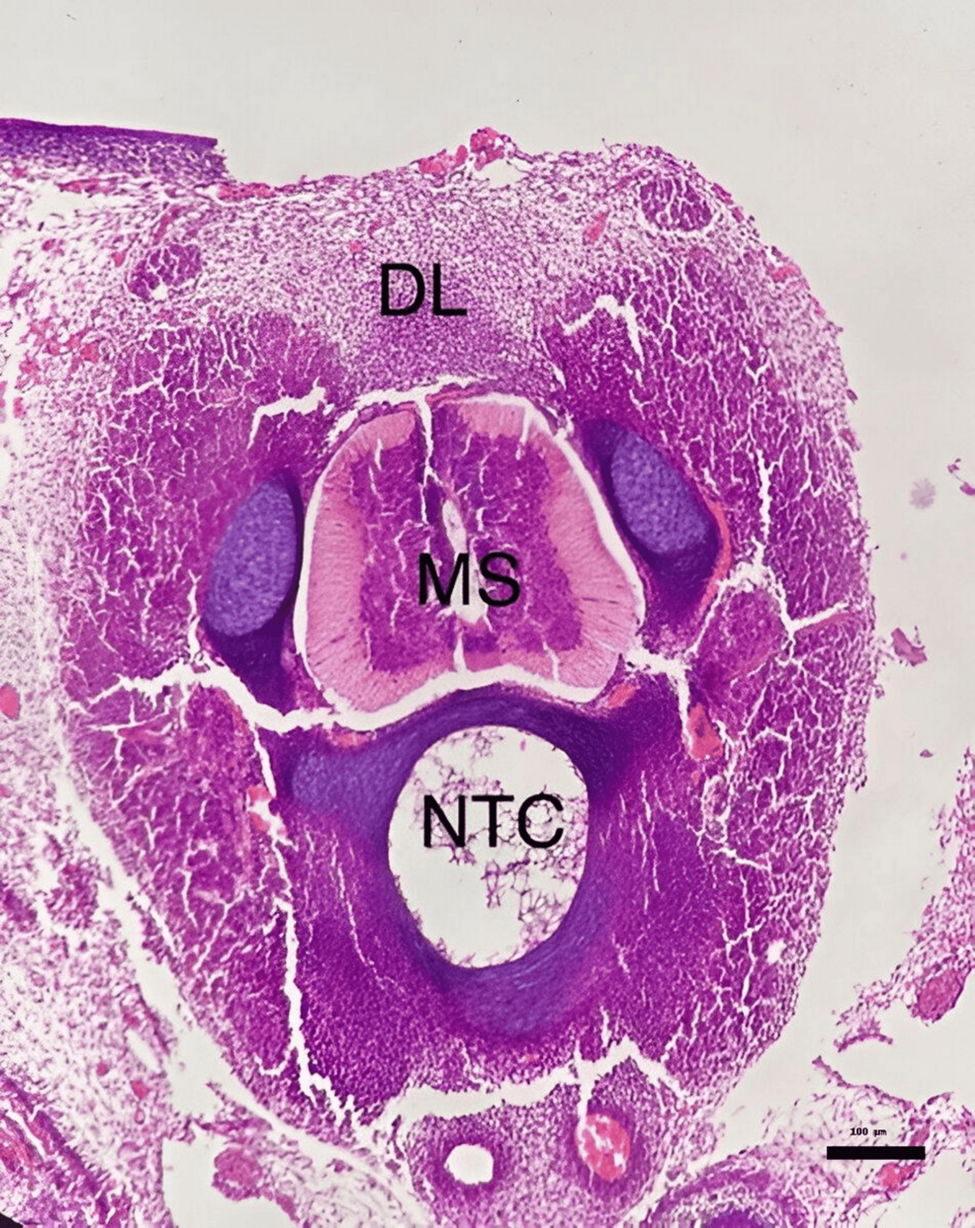
Defective lamina and presence of an open neural tube in a pantoprazole-treated embryo (0.4 mg/kg). Histological section of a chick embryo showing an incomplete midline closure characterized by the absence of fusion between the neural folds and the presence of an open neural canal. (Hematoxylin & Eosin stain, ×100 magnification.) (DL: Defective Lamina, MS: Medulla Spinalis, NTC: Notochord)

**Figure 2:**
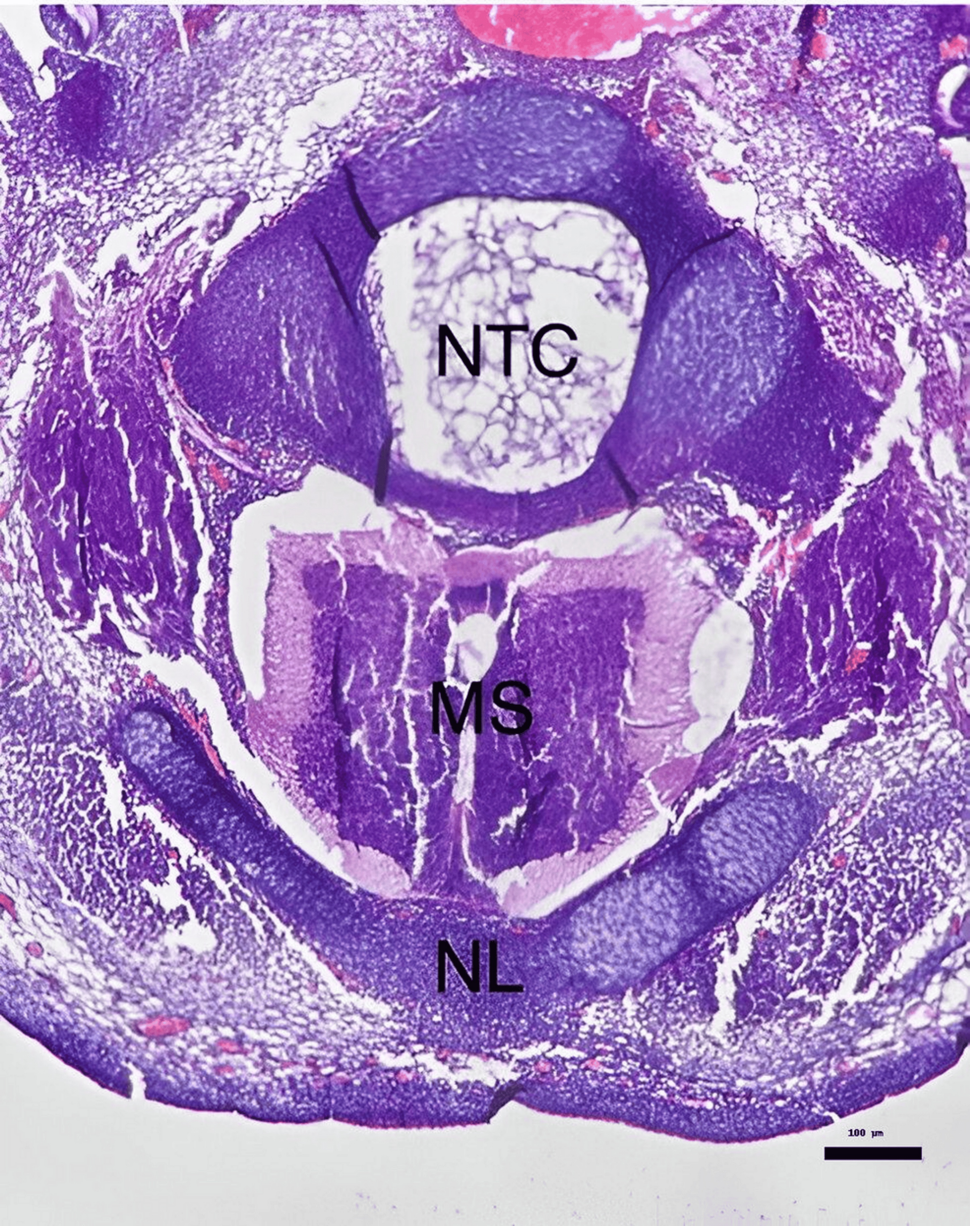
Closed neural tube and normal embryonic development in a pantoprazole-treated embryo (0.2 mg/kg). Histopathological image demonstrating a fully closed neural tube (NTC) with intact neural lamina (NL) and well-organized medulla spinalis (MS). (Hematoxylin & Eosin stain, ×100 magnification.)

Neural tube opening was not observed in the control, vehicle, or low-dose pantoprazole (0.2 mg/kg) groups. However, one embryo in each of the 0.4 mg/kg and 0.8 mg/kg pantoprazole groups exhibited an open neural tube defect, while the remaining embryos demonstrated incomplete or delayed closure (Figures 1-2). Statistical analysis showed no significant difference among groups (p = 0.548), but the occurrence of neural tube patency exclusively in the higher-dose groups suggests a possible dose-related trend toward impaired midline closure.

## Discussion

In this study, the effects of pantoprazole on early chick embryo development were examined, with particular focus on its impact on midline closure. Pantoprazole is a proton pump inhibitor (PPI) that exerts therapeutic effects by inhibiting gastric acid production. However, the results of this study suggest that high doses of pantoprazole may have toxic effects on embryonic development.

This study demonstrates that exposure to high doses of pantoprazole has adverse effects on embryonic development and increases early mortality. In particular, the early loss of five embryos in the high-dose group (Group V) suggests that pantoprazole reduces embryonic survival and that this effect is dose-dependent. Furthermore, the observation of developmental delays suggests that pantoprazole is a potential risk factor that may slow the embryonic development process. The findings suggest that the effects of high doses of proton pump inhibitors on embryonic development should be investigated more extensively and the molecular mechanisms mediating this process should be examined in detail in future studies. In a study conducted by Nyaga et al. (2023) to determine the effects of prenatal exposure to different doses of pantaprazole on maternal and fetal outcomes in albino rats, it was found that high doses of pantoprazole caused a decrease in mean maternal weight gain, a decrease in the number of offspring, an increase in the number of resorbed endometrial glands and an increase in the number of eaten fetuses [15]. Matok et al. reported that the use of PPIs in the third trimester of pregnancy did not increase the risk of perinatal mortality [16].

Differences in developmental parameters between Group I (control) and the other groups highlight the negative effects of pantoprazole on development. In Group I (control), embryonic length, weight, and head diameter, as key developmental indicators, reflect healthy development. However, compared to Group II (vehicle group) and more notably Groups III, IV, and V (pantoprazole-treated groups), there was a significant decrease in these parameters. Particularly in Group V, developmental delays were more pronounced, and embryonic parameters deviated further from the norms. These findings suggest that pantoprazole, especially in high doses, may have harmful effects during the critical period of embryonic development. Matok et al. reported that the PPI exposure in the third trimester of pregnancy did not cause a significant change in birth weight [16].

In terms of spinal cord development, no statistically significant difference was observed. However, some embryos in Groups IV and V showed incomplete neural arches. The deficiency of neural arches suggests that pantoprazole may have an adverse effect on central nervous system development. This finding indicates that pantoprazole could potentially affect neurological development [17]. However, the lack of statistical significance in this finding suggests the need for a larger sample group and more advanced investigations to confirm this effect.

In this study, it was observed that the maximum recommended daily dose of pantoprazole (70 mg/day) resulted in a statistically significant increase in fetal weight, and a considerable increase in head diameter. This finding warrants additional studies. The differences observed in these studies were attributed to variations between animal models. A review of the literature revealed that the effects of pantoprazole varied [18].

Limitations of the study and future research

While this study has made a significant step toward understanding the toxic effects of pantoprazole on embryonic development, it has several limitations. First, the sample size used was limited. Repeating the study with larger sample groups will help obtain more reliable results. Additionally, considering variations in dosage and duration will be crucial in more deeply investigating the effects of pantoprazole on embryonic development. Future studies should employ different biomarkers and molecular analysis techniques to explore the effects of pantoprazole on embryonic development in greater detail.

## Conclusions

The findings of this study suggest that pantoprazole, particularly in high doses, has significant adverse effects on embryonic development. Developmental retardation, early mortality, and neurological development disorders are indicative of the potential toxic effects of pantoprazole on embryonic development. It has been observed that pantoprazole, especially at high doses, can cause developmental. These findings highlight the need for further investigation into the toxic effects of pantoprazole on embryonic development.

As a result of the study, it was determined that pantoprazole caused developmental delays. The effects of pantoprazole on the fetus were found to be dose-dependent. Examination of Table 2 revealed that physical measurements increased with higher doses.
